# Outdoor occupational environments and heat stress in IRAN

**DOI:** 10.1186/s40201-015-0199-6

**Published:** 2015-05-28

**Authors:** Hamidreza Heidari, Farideh Golbabaei, Aliakbar Shamsipour, Abbas Rahimi Forushani, Abbasali Gaeini

**Affiliations:** Department Occupational Health, School of Public Health, Tehran University of Medical Sciences, Tehran, Iran; Department Physical Geography, School of Geography, University of Tehran, Tehran, Iran; Department Epidemiology and Biostatistics, School of Public Health, Tehran University of Medical Sciences, Tehran, Iran; Department Sport Physiology, School of Physical Education and Sport Science, University of Tehran, Tehran, Iran

**Keywords:** Outdoor worker, Heat stress, Climate change

## Abstract

**Background:**

The present study aimed at demonstrating the heat stress situation (distribution and intensity) based on a standard and common heat stress index, Wet Bulb Globe Temperature (WBGT), during hot seasons and interpret the obtained results considering global warming and rising temperature in different parts of the country based on climate changes studied in Iran.

**Methods:**

Heat stress assessment was done using WBGT index. Environmental parameters were measured simultaneously in the early, middle and end of shift work. The personal parameters including cloth thermal insulation and metabolic rate of 242 participants from 9 climatic categories were recorded for estimating effective WBGT (measured WBGT plus cloth adjustment factor as well as metabolic rate effect). The values of the indicator were categorized in the statistical software media and then linked to the climatic zoning of the data in the GIS information layers, in which, WBGT values relating to selected stations were given generalization to similar climatic regionalization.

**Results:**

The obtained results showed that in the summer about 60 % and more than 75 % of the measurements relating to 12 pm and 3 pm, respectively, were in heat stress situations (*i.e.* the average amount of heat stress index was higher than 28 °C). These values were found to be about 20–25 % in the spring. Moreover, only in the early hours of shift work in spring could safe conditions be seen throughout the country. This situation gradually decreased in the middle of the day hours and was replaced by the warning status and stress. And finally, in the final hours of shift work thermal stresses reached their peaks. These conditions for the summer were worse.

**Conclusions:**

Regarding several studies related to climate change in Iran and the results of present study, heat stress, especially in the central and southern parts of Iran, can be exacerbated in the decades to come if climate change and rising temperature occurs. Therefore, paying attention to this critical issue and adopting macro-management policies and programs in the field of workplace health is essential.

## Background

The Islamic Republic of Iran lies in western Asia. The population now estimated at 72.0 million. The area coverage of different types of climate in Iran is 35.5 % hyper-arid, 29.2 % arid, 20.1 % semi-arid, 5 % Mediterranean and 10 % wet (of the cold mountainous type). Thus more than 82 % of Iran’s territory is located in the arid and semi-arid zone of the world [1]. Weather and climate play a significant role in people's health. Changes in climate affect the average weather conditions to which we are accustomed. Warmer average temperatures will likely lead to hotter days and more frequent and longer heat waves. This, in turn, could increase the number of heat-related illnesses and deaths [2].

In 2009, the U.S. Global Change Research Program presented a report to congress that summarizes current and future impacts of climate change on the U.S., including how it directly affects humans. As extreme heat waves become more common, there is an increased risk of heat-related illness and death. The elderly, the very young and diabetics face the highest threat [3].

The impacts of climate change on health will depend on many factors. These factors include the effectiveness of a community’s public health and safety systems to address or prepare for the risk and the behavior, age, gender, and economic status of individuals affected. Impacts will likely vary by region, the sensitivity of populations, the extent and length of exposure to climate change impacts as well as society’s ability to adapt to change. If high temperatures, especially when combined with high relative humidity, persist for several days (heat waves), and if nighttime temperatures do not drop, extreme heat can be a killer. Of all climate-related projections by scientists, rising temperatures are the most robust [4]. Climate change can affect problems that are already huge, largely concentrated in the developing world, and difficult to combat. WHO and its partners are devising a research agenda to get better estimates of the scale and nature of health vulnerability and to identify strategies and tools for health protection [5]. Researches done in the Iran show that similar the other parts of the worlds, human activities either directly or indirectly cause an increase in most greenhouse gas concentration [6, 7]. They predict this rise may increase greenhouse effect and create a warmer planet. Based on general circulation models (GCM) and scenarios used by Intergovernmental Panel on Climate Change (IPCC) [8], it is anticipated that global temperature in the 2100 s will be warmer than that in the 1900s between 1.1 and 6.4 °C [9]. New climate models also predict that average global temperatures over the range of 1.4–5.8 °C will increase in this century [10]. In Iran some researchers have investigated changes and trends in temperature and precipitation in different regions. Almost all of them find that an increase in air temperature in the decades to come on the other hands, this increase will be more intense in some areas of the south and central regions than the northern areas [6, 7]. The rate of increase in the temperature of 2025 onwards shows higher figures. In the case of reduction of precipitation the results obtained have been roughly the same. Although most studies conducted in different parts of the world are about climate change and the environmental impact of this phenomenon [11–14], in terms of occupational health and the effects of global warming on workers’ health, especially workers in outdoor environments has received less attention [15]. Global warming can produce a lot of health problems due to occupational heat exposure [16], which the most of them can be creating heat stress and diseases for all people especially those who work in hot environments. Workers at the risk of heat stress include outdoor workers (who define as any worker who spends time outside) and workers in hot environments such as fire fighters, bakery workers, farmers, construction workers, miners, boiler room workers, factory workers, and others. Workers at the greater risk of heat stress include those who are 65 years of age or older, are overweight, have heart disease or high blood pressure, or take medications that may be affected by extreme heat. Outdoor workers are exposed to many types of hazards depending on their type of work, geographic region, season, and duration of time they work. Extreme heat conditions can cause heat stroke, heat cramps, heat exhaustion, heat rash, and other problems such as loss of productivity and ability to work [17]. The incidence of heat stroke is unclear; it is due to the lack of accurate statistics on referring patients to medical centers and home treatment. Heat stroke incidence in women and men has been reported alike, but in the case of men in our country -because of their involvement in a position which contributes to heat stroke- the incidence rate has been higher. Tropical diseases in warm seasons are regarded as common diseases in the world. They are frequently seen in our country, especially in hot, dry, warm and moist areas. Therefore awareness of the current status of thermal stress in different parts of the country as well as climate change trends in the future in each area is necessary for taking preventive measures and improving the occupational environments. In spite of the importance of global warming and its adverse health effects on human beings, unfortunately there are a few studies from the viewpoint of occupational health of workers in outdoor workplaces. The Impact of global warming is a growing concern and there is a need for regional-based studies to quantify the degree of stress due to thermal environment. For over a century attempts have been made to construct an index to describe heat stress satisfactorily so that between the years 1905 and 2005 approximately 40 heat stress indices have been provided [18]. Some of them are widely used and general with simple use which can produce appropriate estimations for heat stress. Because of larger number of workers occupied in outdoor environments and existence of different climatic regions in Iran ranging from very hot and dry to very hot and humid regions, it is important to perform a comprehensive investigation to reveal low, moderate and high risk areas considering heat stress. This is important for taking preventive and control measures of regulatory health agencies. As such, the aim of the present study is to demonstrate the heat stress situation (distribution and intensity) based on a standard and common heat stress index, Wet Bulb Globe Temperature (WBGT), during hot seasons and then interpret the obtained results considering rising temperature in different parts of the country based on climate changes studied in Iran.

## Methods

In order to reach the aim of the study, that is, to evaluate heat stress in outdoor environments, DeMartonne climate classification was used after a few modifications for Iran. The classification is determined by the 14 climatic zones, including hot and dry, hot and humid, temperate, cold and dry, cold and moist, with different intensities determined. Due to the nature of research that involves the heat stresses, only 9 of 14 above-mentioned categories were selected (Table 1). Cold regions were not included in these 9 climate categories.242 acclimatized subjects from nine climatic regions who worked in outdoor occupations, including railroad workers, farmers, shipbuilding, steel, cement and asphalt industry, *etc.* have been participated in this investigation and their individual heat exposure were assessed. All of them were considered acclimatized and worked at least 6–7 h per day (75 % work plus 25 % rest each hour). To assess heat stress we used WBGT index and corrected it for cloth thermal insulation and metabolic rate of each subject. It means that not only measured WBGT (using environmental parameters) was used in this study, but also effective WBGT was estimated by correction for clothing adjustment factor (CAF), as well as activity level or metabolic rate, two important personal parameters, which should be considered when individual heat stress exposure assessment is necessary. The clothing adjustment factor and metabolic rate were estimated according to ISO-9920 and ISO-8996, respectively for all of 242 participants and then corrections were made to achieve effective WBGT, if necessary.

**Table 1:** Description of studied climate categories

Nominal category^a^	Climate category	Synoptic stations
Arid, cool and warm to very warm region	Site 1	Tehran, Semnan, Qazvin, Arak, Qom
Semi arid, moderate and very warm region	Site 2	Ilam, Ahvaz
Semi arid, cool and warm region	Site 3	Bojnourd, Zanjan, Shiraz, Mashhad
Arid, cool and warm region	Site 4	Birjand, Kerman
Arid, cool and very warm region	Site 5	Esfahan, Zahedan, Yazd
Arid, moderate and very warm region	Site 6	Bushehr, Chabhar, Bandarabbas
Humid, cool and warm region	Site 7	Noshahr, Ramsar, Sari
Semi Humid, cool and warm region	Site 8	Gorgan, Yasouj, Ghasre-Shirin
Post Humid, cool and warm region	Site 9	BandarAnzali,rasht

^a^Cool in the nominal category is one of the properties of winter weather conditions, not necessarily spring and summer weather conditions which is emphasized on in this study

The WBGT index is by far the most widely used heat stress index throughout the world. The heat effects are dependent upon not only air temperature, but also humidity, air movement, radiated heat, clothing, individual ability to sweat and the workers’ physical activity level (metabolic rate). A commonly used “heat stress index” that combines the influence of the four environmental factors into one number is the WBGT [17]. It was developed in a US Navy investigation to heat casualties during training [19] and adopted by ISO-7243 [20] as an approximation to the more cumbersome corrected effective temperature (CET), modified to account for the solar absorptive of green military clothing. It is given for conditions with solar radiation, outdoor areas, as follows: [21]:

$$ \mathrm{WBGT}=0.7\mathrm{t}\mathrm{n}\mathrm{w}+0.2\mathrm{t}\mathrm{g}+0.1\mathrm{t}\mathrm{a}, $$

Where:

tnw
  the natural wet bulb temperature
tg
  the temperature of a 150 mm diameter black globe
ta
  is the air temperature.

The index has been adapted by the World Health Organization (WHO). In 1982, it was approved by the ISO organization as an international standard for heat load assessment. In 1986, the National Institute for Occupational Safety and Health (NIOSH, 1986) set it as the criterion for occupational exposure to a hot environment. Later on, work-rest regime regulations were made based on this index [22]. In this research WBGT index was determined using the Heat Stress Monitor (Casella Microtherm WBGT, UK). Environmental parameters including air temperature, globe temperature, natural wet temperature, air velocity and relative humidity were measured simultaneously in three interval periods of shift work at 9 am, 12 pm and 3 pm corresponding to early, middle and end of shift work, respectively. In order to have a wide range of weather conditions, all measurements were evaluated twice a year, once in spring and once in summer. To generalize the studied stations to similar climatic regions in country, at first, the measured values of WBGT index for each station were linked to similar climatic stations. The values of the indicator were categorized in the statistical software media and then linked to the climatic zoning of the data in the GIS information layers, in which, WBGT values relating to selected stations were generalized to similar climatic regionalization. Therefore, in this process the spot information were converted to regional information.

For generalization of the data to the other similar climatic regionalization, the long-term data of each weather station (between 1965 and 2009) including daily minimum and maximum temperature, precipitation rate and sun shine were utilized. Finally, according to the ISO-7243 and ACGIH [23] reference values (Tables 2 and 3), three ranges including safe, caution and stress were assumed for less than 25, 25–28 and more than 28° of centigrade, respectively. In the selection of these values, all workers were supposed to be acclimatized and have moderate work load. The results of heat stress situations in spring and summer as well as interval periods of a shift work were presented graphically on several maps of Iran using Arc/GIS 10.2. Finally according to the obtained results, some recommendations and preventive measures of heat stress were offered.

**Table 2:** ISO 7243: WBGT reference values

Metabolic rate (W.m^−2^)	WBGT reference values	
	Acclimatized (°C)	Not acclimatized (°C)
Resting M < 65	33	32
65 < M < 130	30	29
130 < M < 200	28	26
200 < M < 260	25 (26)^a^	22 (23)^a^
M > 260	23 (25)^a^	18 (20)^a^

^a^Figures in brackets refer to sensible air movement

**Table 3:** WBGT threshold limit values (°C)

Work- rest regimes	Work load		
	Light	Moderate	Heavy
Continuous work	30.0	26.7	25.0
75 % work + 25 % rest; each hour	30.6	28.0	25.9
50 % work + 50 % rest	31.4	29.4	27.9
25 % work + 75 % rest	32.2	31.1	30.0

From ACGIH (American Conference of Governmental Industrial Hygienists, 2013)

## Results

As mentioned above, after WBGT index was measured, it was corrected for thermal insulation and work load to reach effective WBGT. The thermal clothing insulation of the subjects was ranging from 0.45 to 1.52 Clo (Mean ± SD equal to 0.79 ± 0.16 Clo) and for the case of metabolic rate, it was ranging from 115 to 230 W.m^−2^ (Mean ± SD equal to 179.49 ± 23.36 W.m^−2^). These amounts of work load indicate moderate to heavy works. Environmental parameters measured for work stations of 242 outdoor workers during spring and summer at 9 am, 12 pm and 3 pm are presented in Table 4.

**Table 4:** Range of environmental parameters in this study

Environmental parameters	N^a^	Min	Max	M ± SD
Air pressure (mmHg)	1452	594.9	775.6	705.98 ± 57.19
Relative humidity (%)	1452	20.9	93.8	51.78 ± 16.86
Dry air temperature (°C)	1452	14.6	46	31.63 ± 6.18
Globe bulb temperature (°C)	1452	16	49.3	35.09 ± 6.78
Natural wet temperature (°C)	1452	13.2	32.5	22.98 ± 4.29
Air velocity (m/s)	1452	0.02	7.25	1.27 ± 1.35

^a^This number of measurements resulted in 242 work stations by repetitions of 3 times in a day and twice in a year (Spring and summer at 2013)

As noted in Table 4 we can see the range of environmental parameters measured is very broad, so, for example, the dry air temperature parameter has a range from 14.6 to 46° and relative humidity has values between about 21 to the 94 %. In the case of other environmental parameters, a similar situation can be seen. These values, which have been obtained from different parts of the country in spring and summer, confirm the existence of very diverse climatic conditions in the country.

The analysis of covariance in Generalized Linear Model showed that the effect of climatic category, season and time as well as the interaction of these factors is significant in less than 0.001.

**Fig. 1:**
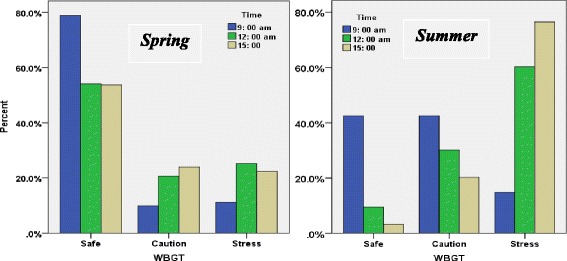
Thermal condition according to WBGT classification in three times and thermal categories

As shown in Table 5, based on reference value of the WBGT (28 °C for moderate work load and acclimatized workers) a number of measured stations have a higher average of the maximum limit, in which these conditions are much more abundant in the summer than in the spring. On the other hand in the sites 5 and 6 corresponding to the areas of central and south of the Iran, inappropriate thermal situation was observed in both the spring and summer. The diagrams of Fig. 1 show thermal stress condition in the spring and summer, and in three thermal situations including safe, caution and stress on the basis of what was described in the methods section. These charts which are also provided for different work shift hours show that between 12 and 3 pm (the middle to end hours of work shift) heat stress state is notable, so that in the summer about 60 % and more than 75 % of the measurements which are related to 12 pm and 3 pm, respectively, were in heat stress situations. These values were found to be between about 20 and 25 % in the spring.

**Table 5:** Mean and standard deviation of WBGT_effc_ in spring and summer in different sites and times of shift work

Climate category	Time	WBGT_effc_ (°C)	
		Spring M ± SD	Summer M ± SD
1	9 AM	18.94 ± 2.98	24.30 ± 0.38
	12 PM	21.52 ± 1.50	27.20 ± .54
	3 PM	22.64 ± 1.18	29 ± 0.51
2	9 AM	24.01 ± .97	27.05 ± 1.12
	12 PM	26.20 ± 2.40	31.20 ± 1.35
	3 PM	27.17 ± 2.57	32.97 ± 1.98
3	9 AM	21.72 ± 2.14	25.48 ± 0.63
	12 PM	26.03 ± 1.27	27.38 ± 2.46
	3 PM	25.44 ± 1.13	29.38 ± 1.18
4	9 AM	18.60 ± 1.76	23.55 ± 2.26
	12 PM	19.91 ± 1.62	25.18 ± 0.29
	3 PM	19.84 ± 1.33	25.78 ± 0.99
5	9 AM	27.12 ± 0.93	24.56 ± 0.48
	12 PM	29.85 ± 0.77	28.12 ± 0.93
	3 PM	30.68 ± 2.05	32.38 ± 0.66
6	9 AM	29.40 ± 0.42	32.55 ± 0.37
	12 PM	31.12 ± 0.15	34.68 ± 1
	3 PM	33.53 ± 1.18	35.10 ± 0.55
7	9 AM	17.50 ± 0.99	25.35 ± 0.70
	12 PM	17.85 ± 2.37	28.86 ± 0.45
	3 PM	18.17 ± 2.45	29.77 ± 0.38
8	9 AM	22.90 ± 0.67	27.79 ± 1.34
	12 PM	23.78 ± 0.23	30.84 ± 1.68
	3 PM	23.32 ± 1.18	31.59 ± 0.48
9	9 AM	22.30 ± 0.50	26.04 ± 1.04
	12 PM	25.69 ± 1.20	27.60 ± 2.20
	3 PM	25.18 ± 2.29	28.16 ± 1.95

**Fig. 2:**
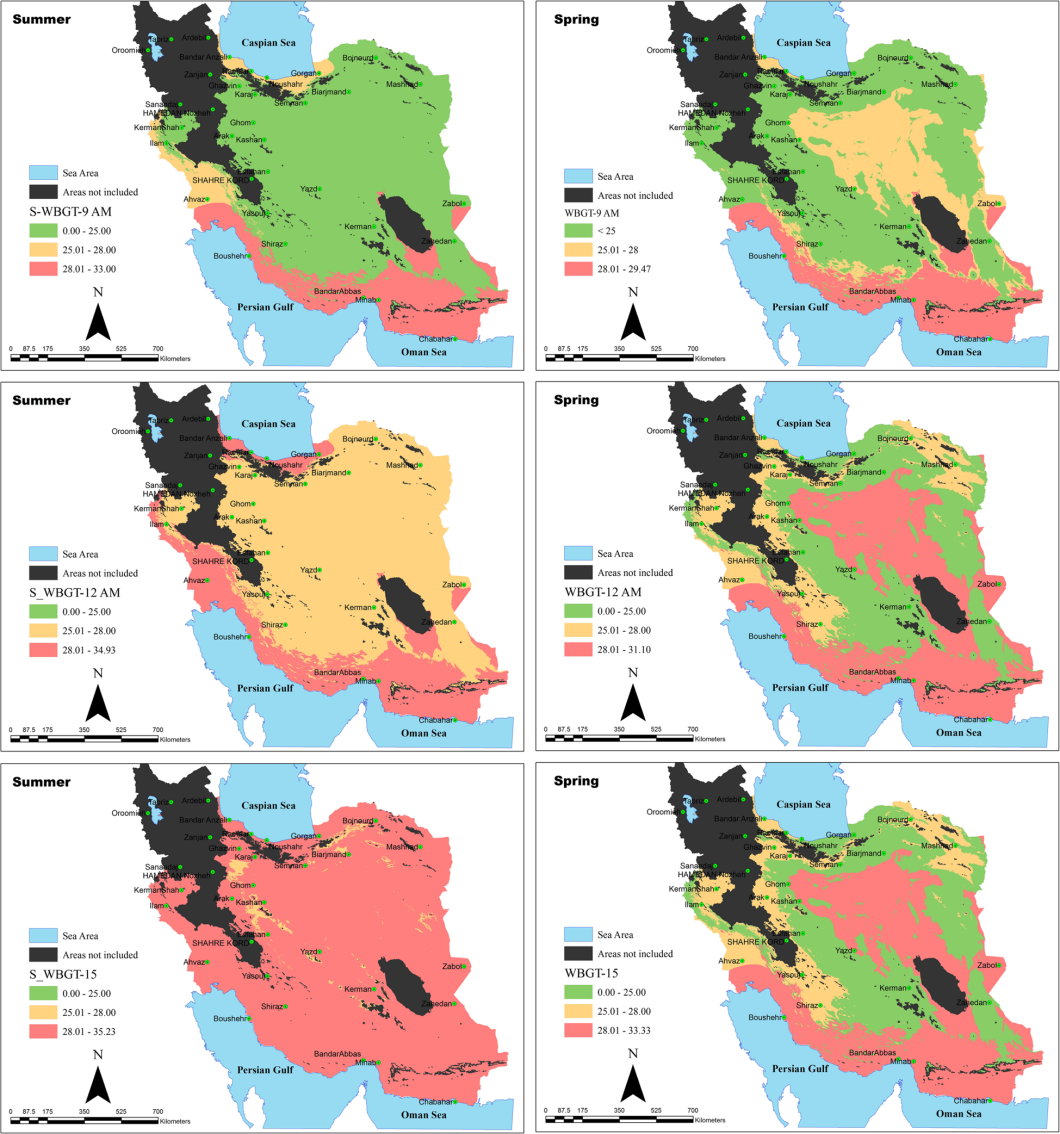
Assessment of thermal situation based on changes of mean WBGT index in spring and summer during a shift work

In order to clearly show thermal stress situation throughout the country, after the data connection of the measuring stations to the stations with a similar climate and generalizing the results throughout the country using Arc/GIS 10.2. program, thermal condition in both spring and summer, as well as at different hours of the day [9, 12, 15] and on the three thermal regions including safe, caution and stress areas were shown in six graphic maps separately (Fig. 2). As is clearing the maps, only in the early hours of shift work in the spring can safe conditions be seen throughout the country. This situation gradually lessens in the middle of the day hours and is replaced by the warning status and stress. And finally, in the final hours of work shift thermal stresses reach their peaks. These conditions, as specified in the maps for the summer, are even worse. So in the final hours of the summer almost neither safe nor warning conditions could be seen while working across the country along with extreme thermal stress. Another noteworthy point is that-as the maps elucidate - it is only in the southern areas of the country and to some extent its central parts corresponding to very hot and humid and very hot and very dry regions, respectively, that heat stress condition can be seen in both spring and summer and at all periods of shift work (from the early hours to the final hours of shift work).

## Discussion

This study aimed to assess the status of the thermal stress in the warm seasons of the year in Iran and demonstrate how thermal stress situations may be changed in different climates regarding predicted rising temperature? It focused on temperature rising in different regions of Iran based on local investigations done so far. This assessment was performed based on the very common and standard index, WBGT, indicating thermal stress conditions in three levels of safe, alert and stress areas. Mention can be made of similar studies worldwide. For example, in a study performed from the years 1995 to 2012 over Dum Dum (22°34’N/88°22’E) in the district of North 24 Parganas, West Bengal which falls under suburban climate, the distribution of stressful days of summer months (March, April and May) was determined. In that study thermo-hygrometric index, THI, was used to assess heat stress by estimation from the daily weather data. It has been observed that 72.99 % days of summer months have discomfort scores ranging from 3 to 5. The number of stress events is high in the month of May (59.37 %). Estimated values of WBGT (Wet bulb globe temperature) and RSI (Relative strain index) have shown that both the indices are significantly correlated with THI having R^2^ values 0.95 and 0.91, respectively. Regional based scales of WBGT and RSI are proposed based on the recognition of discomfort scores of THI [24]. The results of this study, as shown in maps of Fig. 2, specify that the maximum thermal stress can be seen in the Centre and South of the country, so that between 12 and 3 pm in the summer, almost any point of the country is not a safe condition, but conditions have to be alert and stressful. This situation in the Center and South of the country has been observed to be higher than that in other areas (sites 2, 5 and 6 in Table 1). In one comprehensive investigation performed in Iran, the whole country was assessed to create climatic classification based on Tourism Climate Index (TCI). The results showed that with the exception of the Northwest Territories (the areas which are not included in our research; because it was assumed these areas were never involved with heat stress and probability of their placement in heat stress region is almost negligible) and in parts of the Northeast, which has favorable status in the summer, almost in the entire country inappropriate conditions can be seen [25]. Therefore, in non- occupational outdoor environments such as pleasure places, sports fields and so on, the heat stress is also the issue which should be paid more attention regarding rising temperature in the future to take control measures. These results are in tandem with the results of the present study, so that more intense heat stress can be seen in the South (the cities of Bandarabbas, Ahvaz, Bushehr) and Centre (the cities of Yazd and Qom) of the country. In Southern regions the WBGT index values were determined during middle and end of work shift higher than 31 °C and 34 °C, for the spring and summer, respectively. In central regions such as Yazd, Esfehan and Qom, the heat stress situation especially for the summer were assessed also higher threshold limit values. The results of the study by Mohammadi et.al on prediction of global warming effects of different areas of Iran yield similar results, which made it clear that in decades to come the country will face an increase in average temperature and that this increase in some areas of the South and Central regions of the country is more noticeable than that of other regions [6]. In another study, Abbasi and Asmari [26] modeled the climate of Iran and changes in perspiration and temperature using General Circulation Models. The prediction of the used models (ECHAM_4_ and HadCM_2_) showed that on the average, 3–3.6 °C increases in maximum temperature until decade 2100. Considering rising temperature in the future over the entire of the country especially southern and central regions of it, and WBGT index values obtained in this study, it can be concluded that in spite of huge problems related to heat stress can be see already in several parts of the country, the situation will be worse in future decades regarding to rising temperature and climate change in Iran. There are desert and very hot areas with long hours of sun’s radiation in the center of Iran. Moisture is low in these areas and in some sections it is so low that–as is shown in Table 4- moisture level is as low as 20–30 % and the radiant temperatures of about 50° Celsius are the characteristics of these areas. The southern areas of the country which have a desert climate and are very hot, due to the vicinity of the Oman sea and the Persian Gulf, not only experience very high air temperature (36.93 ± 3.00 °C), but also have high moisture conditions (61.19 ± 8.88 %). The combined effect of these two environmental factors plays a vital role in heat stress incidence in these areas. As the geographical maps show, throughout these areas heat stress exposure exists throughout shift hours in hot months, so that the least amount of WBGT average related to 9 a.m. in the spring and its maximum average is related to the 3 pm in the summer. These values were obtained to be 29.40 ± 0.42 and 35.10 ± 0.55 for minimum and maximum average, respectively. It means that the obtained results of mean WBGT index for regions with minimum and maximum heat stress are, respectively, 1.4 ± 0.42 and 7.1 ± 0.55 °C higher than those of the threshold limit value presented by ACGIH. In another study done in the Persian Gulf weather conditions during hot seasons, the mean (SD) of WBGT values was 33.1 (2.7). The WBGT values exceed those of American Conference of Governmental Industrial Hygienists (ACGIH) standard in 96 % of work stations [27].

In the northern regions of the country (sites 7 and 8) due to the vicinity to the Caspian Sea on the one hand, and the existence of the Alborz Mountains with a little distance from the sea on the other hand, there are highly humid conditions (70.36 ± 11.22). But fortunately, in the spring due to the relatively low average air temperature (25.38 ± 6.59), heat stress usually cannot be seen even in the middle and final hours of shift work. The situation will change in the summer when warming air is combined with high humidity, so, as shown in Table 5, in the middle and final hours of shift work WBGT index is higher than the limit allowed. This is the case which has been well illustrated in maps of Fig. 2. This is compatible with the results of [25]. The remarkable thing found in the current study is that the basis of assessing the situation of thermal stress is the amount of 28 °C reference value, excess of which may bring about heat stress condition. This amount for action limit has been assumed to be between 25 and 28 °C. Clothing type and amount of thermal insulation, different metabolic rates and work- rest regimens, as well as having protective devices can influence the WBGT reference value. Also, many of workers, particularly workers with low working experience may not be acclimatized. Therefore, with regard to these considerations and the fact that outdoor works in Iran are less organized and health supervision such as provision of plenty of cool and healthy water, regular work and rest cycles, proper shelter to rest may not be sufficient for them, thermal stress status can be more troublesome and will require special attention.

## Conclusion

Heat exposure based on WBGT index exceeded threshold limit value in entire the country in the summer especially between 12 and 3 pm. The south, west south, east south and large areas of central regions of Iran experienced very hot situation, not only in the summer, but also in the spring.

With regard to the status of the existing and projected thermal stress in relation to air temperature increase, especially in the central and southern parts of Iran, workers especially who work outdoor will be encountered with more heat stress in the decades to come.

There is a great need for paying due attention to this critical issue and adopting macro-management policies and programs in the field of occupational health.
